# Quarantine in Diphtheria

**Published:** 1881-04

**Authors:** 


QUARANTINE IN DIPHTHERIA.

Dr. A. T. Conley, of Cannon Falls, Minnesota, writes to the *Medical Record*, February 19: Our village and surrounding country have just passed through the worst epidemic (of diphtheria) ever known in these parts.

After careful study of the disease I have become convinced that we cannot find the specific cause of diphtheria in the effluvia of decaying vegetable or animal matter, conveyed either through air or water, for this reason: We have had, so far, a much more than ordinarily severe winter, not a single thaw since the last of October, the mercury most of the time below zero, and yet, perhaps, Minnesota never suffered so severely from diphtheria. I believe medical men will agree with me when I say that the most malignant epidemics of diphtheria are to be found in the Northern States *and in the winter.*

How can this be accounted for by the advocates of the theory that the specific cause can be found in the decomposition of vegetable or animal matter, when no such decomposition is taking place? There may be such a thing as spontaneous diphtheria, but this I think not more likely than spontaneous small-pox. I believe, for children, diphtheria is as contagious as small-pox.

As soon as diphtheria appeared in our vicinity, we had a competent sanitary committee appointed who visited every house and examined into all sources of filth, and our village was put into the best sanitary condition possible, and yet the disease has carried off twenty-two of our children in the dead of winter. I have further noticed that filthy families have suffered no more than the cleanest; that families who lived in the immediate vicinity of manure piles suffered no more than those who were far removed from such sources of contamination.

I believe we have only succeeded in getting rid of the plague by the strictest quarantine, closing all schools and churches, and prohibiting public gatherings of all kinds, and allowing only physicians and nurses to visit the sick ; the village paying a man to take orders and deliver everything needed by families where the disease existed, at the gate, and after the disease subsided, thoroughly disinfecting and cleaning up. In short, treat it the same as small-pox. I believe I can trace every case, and account for it by contagion solely, in our village.

Now, while I am a strict believer in the laws of hygiene, and that cleanliness is next to godliness, and that towns and cities cannot be too strict in enforcing all laws for the removal of all sources of filth, still they must go farther in dealing with diphtheria, and enforce strict quarantine, and the sooner medical men are united on this point the sooner will diphtheria lose much of its formidableness.

